# Bioequivalence of Two Intravenous Artesunate Products with Its Active Metabolite Following Single and Multiple Injections

**DOI:** 10.3390/ph4010138

**Published:** 2011-01-07

**Authors:** Qigui Li, Lisa Xie, Victor Melendez, Peter Weina

**Affiliations:** Division of Experimental Therapeutics, Walter Reed Army Institute of Research, Silver Spring, MD 20910, USA

**Keywords:** intravenous artesunate, dihydroartemisinin, bioequivalence, bioavailability, rats

## Abstract

In animal species and humans, artesunate (AS) undergoes extensive and complex biotransformation to an active metabolite, dihydroartemisinin (DHA). The bioequivalence of two intravenous AS pharmaceutical products with 5% NaHCO_3_ (China Formulation) or 0.3 M PBS (WRAIR Formulation) was determined in rats in a two-formulation, two-period, and two-sequence crossover experimental design. Following single and multiple intravenous administrations, a series of blood samples was collected by using an automated blood sampler and drug concentrations were analyzed by LC-MS/MS. The 90% CI of the difference between the two intravenous formulations was contained within 80–125% of the geometric mean of pharmacokinetic parameters for AS and DHA in all animals dosed. Hematological effects were studied on days 1 and 3 after the final dosing, and a rapidly reversible hematological toxicity (significant reductions in reticulocyte levels) was seen in the peripheral blood of the rats treated with each formulation. The results showed that bioequivalence with the parent compound and active metabolite was fulfilled in the 82.3–117.7% ranges of all parameters (AUC_0−t_, C_max_, concentration average and degree of fluctuation) in the two-period and two-sequence crossover studies following single and repeated intravenous injections. For the metabolite, the equivalence was satisfied in most pharmacokinetic parameters tested due to the variability in the hydrolysis rate of AS to DHA. The WRAIR formulation of AS was considered to be bioequivalent to the Chinese formulation at steady-state according to the total drug exposure, in terms of both parent drug and active metabolite, rapidly reversal in reticulocyte decline, and extension of single and multiple administrations. Therefore, the parent drug and active metabolites should play similar important roles in the determination of efficacy and safety of the drug.

## 1. Introduction

Injectable artesunate (AS) produced in China is currently used effectively and widely available in much of the World for treatment of severe malaria, and is undergoing the WHO pre-qualification process [1]. However, currently intravenous quinine or quinidine, which have many serious potential adverse effects [2], are the only approved products available for this indication. WRAIR chose to develop an intravenous AS with Good Manufactory Practices (GMP) compliance to provide a potential FDA-approved replacement for current agents used for treatment of severe and complicated malaria.

The effectiveness of AS has been attributed to its rapid and extensive conversion to dihydroartemisinin (DHA), which is therapeutically equivalent to AS and 3-5 fold more active than other artemisinin derivatives [3-6]. Compared to quinine or quinidine, there was no cardiotoxic response seen in beagle dogs when dosed with up to 50 mg/kg of AS for 14 days (our unpublished data). That dose is more than twenty times higher than the anticipated dose to be used in humans. Incubation of AS with malaria parasites *in vitro* can completely inhibit parasite growth within 2–4 h, and it is the artemisinin derivative with activity against all asexual blood stage parasites [7]. When compared to other artemisinins and traditional antimalarial drugs, intravenous AS is the fastest killer in clinical trials, requiring only 3.18 h for parasitemia to fall by half in these patients [8,9]. One problem with the Chinese formulation of intravenous AS is that it has limited stability when dissolved for administration in 5% NaHCO_3_ [10,11].

The pharmacokinetic (PK) parameters of the Chinese AS product for intravenous injection in animal species and humans are very well known. In animals, artesunate has a short half-life and is rapidly converted to DHA [12,13]. Detailed PK data for the Chinese AS and its active metabolite DHA have been reported in adults and children with malaria [4,5], or in healthy volunteers [14]. AS is a highly variable drug with a short elimination half-life (t_1/2_) of 2–18 min and its active metabolite, DHA, has a longer t_1/2_ of 40–60 min [8,9]. The conversion of AS to DHA in humans is much more efficient than in animal species. The ratio of AUC_DHA_ to AUC_AS_ in malaria-infected humans has a range of 4.3–9.7 compared to non-infected rats and dogs with a ratio of only 0.5–0.6 following intravenous injection of AS [5,8].

Chinese injectable AS produced under non-GMP conditions has been used to treat malaria in humans for more than 20 years in many countries throughout the World. In 2003, WRAIR chose to produce a new intravenous AS to take through FDA approval for the treatment of severe and complicated malaria. A new AS injection formulation has been prepared and developed in animal species and clinical trials with much more stable characteristics than the Chinese formulation when dissolved in its dosing solution [15,16]. The comparative bioequivalence (BE) study required by the FDA was conducted following multiple intravenous administrations because they are not only two different formulations, with highly variable AS concentrationd in humans to be considered, but also different levels of the active metabolite, DHA, produced from each AS formulation that actually provide the majority of antimalarial activity are also different. Therefore, artesunate could be considered a prodrug that is converted stoichiometrically to DHA [17,18].

The role of metabolites in BE determination has been extensively discussed [19-21] and continues to be a topic of interest, despite the issuance of the FDA Guidance of Bioequivalence in November 2006 [22]. The guidance proposed that metabolites should be used to assess BE when the parent drug cannot be analyzed or presystemic metabolism that contributes meaningfully to the efficacy and/or safety of the drug product. The present study is designed in accordance with FDA guidance on bioavailability and bioequivalence evaluations with the parent compound and active metabolite in two-period and two-sequence crossover studies following single and repeated intravenous injections with parameters of AUC_0−t_, C_max_, concentration average and degree of fluctuation [23]. To establish the bioequivalence, the calculated confidence interval (CI) should fall within a bioequivalence limit (90% CI), usually 80–125% for the ratio of the product averages [23].

## 2. Results and Discussion

As reported in other literatures and research, artesunate seemed to be a prodrug of DHA in our studies. The plasma concentrations of AS and its active metabolite, DHA, were simultaneously measured by using LC-MS/MS. An automated blood sampler (Culex ABS, Bioanalytical Systems, Inc. West Lafayette, IN) was used for rat blood sample collection. Twenty-four rats were randomized to daily intravenous injection of AS for three days in a two-formulation, two-sequence, and two-period study. The formulations were considered bioequivalent if the 90% confidence interval (CI) of the mean difference for each variable between formulations and periods were within 80% and 125% by calculated with WinNonlin software (WinNonlin 5.2, Pharsight Co. Mountain View, CA. USA).

### 2.1. PK Evaluation of AS on Chinese Formulation (5% NaHCO_3_ Solution)

The multiple intravenous PK parameters of the Chinese formulation of AS are reported in Table 1. Plasma drug concentrations could be measured up to 3 h after once daily 20 mg/kg single injection every time, and the dispositions of AS and DHA in period 1 and 2 were rapid and biphasic.

In the PK analysis of period 1, the mean maximum plasma concentration (C_max_) of AS (parent drug) and DHA (active metabolite of AS) were measured at 5 and 15 min and shown to be 4,300 and 3,361 ng/mL, respectively, on day 1. The mean areas under the curve from 0 to 3 h (AUC_0–3 h_) of AS and DHA after injection were 868 and 1,569 ng·h/mL. Both AS and DHA showed short elimination half-lives of 0.40 h and 0.75 h respectively. On day 3, mean C_max_ values of 2,465 and 2,779 ng/mL were observed for AS and DHA, respectively. Mean AUC_0–3 h_ values of 614 and 991 ng·h/mL for AS and DHA were shown following the last injection. Similar short elimination half-lives were also found for AS (0.41 h) and DHA (0.68 h). The mean concentration averages (C_av_) of AS and DHA were 204 and 330 ng·h/mL respectively, and the degrees of fluctuation (DF) of AS and DHA were 1,216% and 860%, respectively, indicating that AS concentration during the 3 days of treatment is less than that of DHA.

The present study further demonstrates that AS seems to be the prodrug of DHA. In Nature the hydrolysis of AS to DHA is very rapid in rats and yields a concentration ratio of DHA to AS of 1.78 during the 3 days of treatment in the period 1 animals. The total mean AUC values from day 1 to day 3 were calculated as 2,206 and 3,825 ng·h/mL for AS and DHA, respectively. In addition, both concentrations of AS and DHA on day 3 are significantly reduced with AUC_D3_ to AUC_D1_ ratios of 0.70 and 0.65 of for AS and DHA; the mean C_max_ values of AS and DHA were also lower on day 3 than that on day 1 with C_max D3_ to C_max D1_ ratios of 0.42 and 0.87 for AS and DHA, respectively (Table 1).

In the PK analysis of period 2, on day 1, the mean C_max_ of AS and DHA were measured at 5 and 15 min, showing values of 4,139 and 2,689 ng/mL, respectively. The mean AUC_0–3 h_ values of AS and DHA after injection were 932 and 1,223 ng·h/mL. Short elimination half-lives were observed for AS (0.43 h) and DHA (0.73 h). On day 3, mean C_max_ values of 2,542 and 2,452 ng/mL were observed at 5 and 15 min for AS and DHA, respectively. Mean AUC_0–3 h_ values of 665 and 905 ng·h/mL were detected, and similar short elimination half-lives were also found for AS (0.39 h) and DHA (0.72 h) on day 3.

The hydrolysis of AS to DHA was again shown to be very rapid in rats and the concentration ratio of DHA to AS is 1.46 during the 3 days of treatments in the period 2 animals. The total mean AUC values from day 1 to day 3 was calculated as 2,383 and 3,206 ng·h/mL for AS and DHA, respectively. In addition, the concentrations of AS and DHA on day 3 are significantly reduced with AUC_D3_ to AUC_D1_ ratios of 0.71 and 0.75 for AS and DHA; the mean C_max_ values of AS and DHA were also lower on day 3 than on day 1 (Table 1).

### 2.2. PK Evaluation of AS on WRAIR Formulation (0.3M PBS Buffer)

The multiple intravenous PK parameters of WRAIR formulation AS in 0.3 M PBS buffer (pH 8.1) solution for six rats in each period are reported in Table 2. Plasma parent drug concentrations could be measured up to 3 h after once daily 20 mg/kg injection in every animal, and the dispositions of AS and DHA in period 1 and 2 were rapid and biphasic.

In the PK analysis of period 1, on day 1, the mean C_max_ values of AS and DHA were measured at time 5 and 15 min to be 4,032 and 3,153 ng/mL, respectively. The mean AUC_0–3 h_ values of AS and DHA after injection were 879 and 1,460 ng·h/mL. Short elimination half-lives were shown for AS (0.41 h) and DHA (0.98 h). On day 3, the mean C_max_ values of 2,431 and 2,812 ng/mL were observed for AS and DHA, respectively. Mean AUC_0–3 h_ values of 611 and 1,101 ng·h/mL were observed following the last injection for AS and DHA. Short elimination half-lives were also found for AS (0.47 h) and DHA (0.90 h). The mean C_av_ values of AS and DHA were 204 and 367 ng·h/mL, and the DF of AS and DHA were shown to be 1,194% and 765%, respectively, indicating that AS concentration during the 3 days of treatment is significant less than that of DHA.

The hydrolysis of AS to DHA is for the WRAIR formulation is also rapid in rats and the concentration ratio of DHA to AS is 1.77 during the 3 days of treatment in period 1 animals. The total mean AUC from day 1 to day 3 was calculated as 2,244 and 3,869 ng·h/mL for AS and DHA, respectively. In addition, both concentrations of AS and DHA on day 3 are significantly reduced with AUC_D3_ to AUC_D1_ ratios of 0.69 and 0.76 for AS and DHA; the mean C_max_ of AS was also lower on day 3 than observed on day 1 with C_max D3_ to C_max D1_ ratios of 0.70 for the parent drug.

In the PK analysis in period 2, the mean maximum plasma concentrations (C_max_) of AS and DHA were measured and shown to be 4,209 and 3,521 ng/mL, respectively, on day 1. The mean AUC _0–3 h_ values of AS and DHA after injection were 851 and 1,509 ng·h/mL. Elimination half-lives were observed for AS (0.44 h) and DHA (0.91 h). On day 3, mean C_max_ values of 2,710 and 3,124 ng/mL and mean AUC values of 670 and 1,042 ng·h/mL were observed AS and DHA, respectively. The natural hydrolysis of AS to DHA was also shown to be rapid in rats and the concentration ratio of DHA to AS was observed to be 1.70 during the 3 days of treatment in the animals of period 2. The total mean AUC values from day 1 to day 3 were calculated as 2,286 and 3,832 ng·h/mL for AS and DHA, respectively. In addition, both concentrations of AS and DHA on day 3 were shown to be significantly reduced with ratios of AUC_D3_ to AUC_D1_ of 0.78 and 0.71 for AS and DHA (Table 2).

### 2.3. Bioequivalence of AS Injection in Rats

Data collected following the single and multiple intravenous administrations of AS with Chinese and WRAIR formulations in rats were included in the bioequivalence analysis. The main and active metabolite of AS, DHA, was also included in the bioequivalence evaluation.

#### 2.3.1. Bioequivalence analysis after single dose

Bioequivalence evaluation of AS, as a parent drug, was executed in 24 rats following single intravenous administration. The values of the main differences for each variable between the two formulations following single AS intravenous injection in period 1 and 2 were shown in Table 3. For the C_max_ of AS, the 90% CI for the difference between the two i.v. formulations was 90.21–109.79% during period 1 and 2 and the 90% CI was contained within 80–125% of the geometric mean of the C_max_. For the AUC_0–3 h_ of AS, the 90% CI of 88.72–111.28% between the two intravenous formulations was contained within 80–125% of the geometric mean of the AUC_0–3 h_. Thus, bioequivalence criteria for AUC_0–3 h_ and C_max_ for the two intravenous formulations of AS were satisfied in the period 1 and 2 animal studies.

Bioequivalence evaluation of DHA, as an active metabolite of AS, was examined in 12 rats following single intravenous administration. The main difference values for each variable tested for the two formulations following single AS intravenous injection in period 1 and 2 are shown in Table 3. The 90% CI for the C_max_ between the two i.v. formulations was shown to be 75.17–124.78 in the animals of period 1, and this CI was not contained within 80–125% of the geometric mean of the C_max_. However, the 82.04–117.96% CI for AUC_0–t_ was within 80–125% of the geometric mean of the AUC_0-3h_. Therefore, the bioequivalence criteria for AUC_0–3 h_ and C_max_ for the two intravenous formulations of DHA in two periods were outside of the range of bioequivalence.

#### 2.3.2. Bioequivalence evaluation after multiple doses

Bioequivalence evaluation of AS, as a parent drug, was tested in 12 rats following daily intravenous administration for three days. The values of the main differences between the Chinese and WRAIR AS formulations for each variable following multiple AS intravenous injection in period 1 and 2 are shown in Table 3. For the C_max D3_ of AS, the 90% CI for the difference between the two i.v. formulations with 83.68–116.32% during the period 1 and 2, and this 90% CI was contained within 80–125% of the geometric mean of the C_max D3_. For the AUC_D3_ of AS, the 90% CI of the difference between the two intravenous formulations (82.30–117.69%) was contained within 80–125% of the geometric mean of AUC_D3_.

Also, for total 3 days AUC_1–3D_ of AS, the 90% CI for the difference between the two i.v. formulations was shown to be 87.65–112.35% in period 1 and 2, and this 90% CI was contained within 80–125% of the geometric mean of AUC_1-3D_. In addition, for the C_av_ and DF during the 3 days of treatment, the 90% CI for the differences between the two i.v. formulations during the two periods were contained within 80–125% of the geometric mean of C_av_ and DF (Table 3). Thus, bioequivalence criteria for AUC_D3_, AUC_1–3D_, C_max D3_, C_av_, and DF for the two intravenous formulations of AS after multiple administrations were fulfilled in the animals of the two-period studies.

Bioequivalence evaluations of DHA, as an active metabolite of AS, are also shown in Table 3. The 90% CI for the difference between the two i.v. formulations was shown to be 81.94–118.06% for the C_max D3_ of DHA, in animals of the two-periods tested was contained within 80–125% of the geometric mean of the C_max D3_ and AUC_1–3D_. However, the 90% CI of 71.32–128.67% for AUC_D3_ and the 90% CI of 79.11–120.89 for AUC_1–3D_ were not contained within 80–125% of the geometric means of the AUC_D3_ and AUC_1–3D_. The, The 90% CI for the difference between the two i.v. formulations for C_av_ and DF during the 3 days of treatment in two-periods were contained within 80–125% of the geometric mean of C_av_ and DF (Table 3). Therefore, the bioequivalence for C_max_ and AUC on day 3 for the two intravenous formulations of DHA in the two-periods studied were outside of the bioequivalence criteria. Following multiple injections, however, the AUC_1–3D_, C_av_ and DF for the two intravenous formulations of DHA in the two-period did fulfill the established bioequivalence criteria.

### 2.4. Reversible hematological toxicity

After injection of 20 mg/kg of AS intravenously, there were significant reductions in total reticulocyte counts in male and female rats treated with both formulations. On the recovery study, rats treated with 20 mg/kg AS showed significantly hematological changes for most parameters on day 1 after final dosing. There were statistically significant reductions in RBC, Hb and reticulocyte counts in male animals, and reduction in WBC and reticulocyte counts in female rats (Table 4). However, after a short recovery period (3 days post last dosing), all hematological parameters reverted to normal.

### 2.5. Discussion

In the present study, rat blood was collected by using an automated blood sampler, and rat plasma concentrations were analyzed by using a LC-MS/MS. The bioequivalence of the two intravenous formulations was assessed by comparing the mean ratio of C_max_ and AUC_t_.

The standard two-formulation, two-period, and two-sequence crossover design was utilized in this study. According to FDA guidelines, the bioequivalent evaluation was determined followed the requirements to use a non-compartmental PK modeling system [24,25], trapezoidal rule, and 15–20 time points for blood sampling [26]. Formulations were considered bioequivalent if the 90% confidence interval (CI) of the mean difference for each variable between formulations were within 80% and 125% of the two intravenous formulations tested [22].

Although a number of bioequivalent assessments for various drugs were conducted in the rat model [27-29], considerable variability of drug level in population or series time points sampling have been observed in early studies, which has hampered the precision of bioequivalence studies [30-32]. Recently, in spite of the many successful reports of bioequivalence/bioavailability studies in rats by using microdialysis, there are a few challenges that need to be addressed before microdialysis can be regarded as a generally applicable routine technique for assaying drug delivery [33]. During the last decade, automated blood samples have been successfully used to assess series and long team drug delivery [34,35]. The series sampling technique combined with LC-MS/MS has been shown to be minimally invasive and has also been shown to provide PK data with high temporal resolution [36]. In the present study, we combined the two systems for this rat study that has demonstrated the potential for successfully conducting bioequivalence evaluation.

The plasma concentrations of AS and DHA declined during the i.v. treatments in rats. AS concentration was shown to be one-third lower on day 3 compared to day 1, and the AS level observed was similar to the level of DHA, the active metabolite of artesunate, suggesting that an auto-induction of hepatic drug-metabolizing enzymes occurred as a consequence of AS treatment [37,38]. Declining AS drug concentrations have also been reported in humans after AS treatment. Four artemisinin drugs (artemisinin, artemether, AS, and DHA) have shown declining concentrations in plasma after multiple oral treatments in malaria patients and in healthy subjects. The C_max_ and AUC values for AS were markedly reduced by one-third to one-seventh on the last dose day as compared to the first day. The decrease in plasma concentration-time after multiple treatments is indicative of an increase in metabolic capacity due to auto-induction of hepatic drug-metabolizing enzymes [37-39] in patients and in healthy subjects [40].

Artesunate is hydrolyzed almost completely into a single active metabolite, DHA, which has a greater therapeutic effect and, as we have shown in this study, a longer elimination half-life than the parent compound. The role of metabolites in bioequivalence studies has been a contentious issue for many years. A number of papers have published a bioequivalent assessment with only the parent drug or the metabolite [19-21], or recommended the use of metabolite data based on anecdotal evidence from the results of bioequivalence studies [19]. Although it is widely recognized that measurement of metabolite concentrations is crucial to understanding the clinical pharmacology characteristics of a new molecular entity, no clear consensus on the role of metabolites in the assessment of bioequivalence was not within the scientific community was achieved until 1996 [20]. A regulatory policy for the role of metabolites in bioavailability and bioequivalence studies has now been established by the US FDA [22,23].

The FDA guidance proposed that metabolites should be used to assess bioequivalence when (1) the parent is an inactive prodrug; (2) plasma concentrations of the parent drug are too low to monitor due to inadequate assay sensitivity; (3) the parent drug is metabolized rapidly to an active metabolite; and (4) the parent drug and a metabolite both have therapeutic activities but the metabolite is present in higher concentrations. Therefore, in this study, the pharmacokinetic parameters of both the parent drug (AS) and its active metabolite (DHA) were simultaneously monitored for each formulation, and their PK parameters were tested to see whether the 90% confidence interval bioequivalence standard the was met for each parameter. The rat results of this study demonstrated that intravenously administered AS and its active metabolite (DHA) with two different formulations are bioequivalent with respect to exposure following single and multiple doses at steady-state as demonstrated by their similar PK parameters.

## 3. Experimental

### 3.1. Materials and Drugs

Artesunic acid [4-(10′dihydroartemisininoxymethyl) succinate; AS], one of the test agents evaluated, was manufactured as a phosphate salt administered with 0.3 M PBS, and it was obtained from the Walter Reed Chemical Inventory System. It was synthesized and manufactured in GMP compliance by Knoll AG, and rebottled by BASF Pharmaceuticals in Switzerland. The reference AS Chinese formulation compound in this study was manufactured as a sodium salt with 5% NaHCO_3_, and it was manufactured by the Guilin Pharmaceutical II Factory in China. The dosing solutions were prepared within one hour of injection. 5% sodium carbonate for injection was manufactured by the Guilin Pharmaceutical II Factory, Guangxi, China and imported by Atlantic Pharmaceutical Co., Ltd. Bangkok, Thailand. Sterile water, 5% glucose, and normal saline were manufactured by Rhone Merieux, Inc. (Athens, GA).

### 3.2. Test Animals

All Sprague-Dawley rats (young adults; body weight of 186-213 gm) were quarantined (for stabilization) for at least seven days prior to dosing. Rats were individually housed with food and water supplied *ad libitum*. The ethical approval was obtained for the study and all of the bioequivalent studies were conducted in full compliance with the WRAIR Animal Care and Use Committee guidelines. All animal usage, care and handling conformed to “Guide for the Care and Use of Laboratory Animals” [Publication No. 86-23, revised 1996]. Rats were randomly assigned to each dose group prior to dosing, using a randomization program (Excel, Microsoft Office 2007).

### 3.3. Automated Blood Sampler

The Culex ABS is a blood sampling and metabolism monitoring machine, which is controlled by its own internal computer. We were successful in evaluating PK for long-term periods by using this system. After jugular vein cannulation, study animals were individually placed in the system, which allowed for a micro-pump to infuse heparinized-saline (10 unit heparin/mL saline) at a rate of 1-2 μL/min to prevent coagulation within the catheter. Culex samples were obtained with precise blood volumes from the study animals and the total volume taken was less than 6% per day and 14% during the entire study period. Blood samples were maintained at 3 °C using Culex's temperature-controlled fractional collector. Plasma was separated from the blood samples and processed for drug extraction. The detailed sampling process was described previously [15].

### 3.4. Bioequivalence PK in Rats

Replicated crossover designs can be used irrespective of the approach selected to establish bioequivalence, although they are not necessary when an average or population approach is used. Replicated crossover designs are critical when an individual bioequivalence approach is used to allow estimation of within-subject variances for the Test (T) and Reference (R) drug product measures and the subject-by-formulation interaction variance component. The following two-period, two-sequence, and two-formulation design is recommended for replicated bioequivalence studies by the FDA [22] (Table 1).

Three samples were collected during each phase of absorption, distribution, or elimination for full PK analysis on the first and last dosing days following intravenous bonus injection. Additionally, trough levels (pre-dose) samples were collected in between each dose to follow drug accumulation and a series PK approach (18 samples per animal) was used. Thus, each animal was dosed and plasma samples were obtained for up to three days. A total of 18 (0, 5, 15, 30, 45 min, 1, 2, 3, 6, 24.25, 48.167, 48.25, 48.5, 48.75, 49, 50, 51, and 54 h) samples were collected into cooled vials. 50 μL of blood per sample was collected pre-dosing and after dosing and 100 μL per sample was taken from 0.08 h to 56 h after dosing. During collection, blood samples were added into the vials, which contained dried heparin (50 units) on the bottom of each vial. Thus, a total blood volume of 1.75 mL was obtained from each rat during the three day treatment period (0.85 mL of blood on day 0, 0.1 mL on day 1, and 0.9 mL on day 2), which is 9.3% of the animals total blood volume. Subjects for replicated crossover design were followed FDA recommendation [22] with a 2 week wash out period. During wash-out period, if the catheter was blocked up by any reasons the jugular vein tubes were infused or replaced.

### 3.5. LC-MS/MS Assay and Sample Preparation

Standard curves and QC samples were prepared by serial dilutions of neat AS and DHA in plasma after which 100 μL aliquots were transferred to eppendorff vials for extraction by protein precipitation using two volumes of cold acetonitrile. The samples were vortexed vigorously (set at 10 or max setting) for 8–10 sec followed by a 10,000 × g centrifugation at 4 °C for 10 min. Aliquots (approximately 250 μL ea) of the undisturbed supernatants were transferred into a 96-well plate for LC-MS/MS analyses. Rat plasma samples were extracted as described above and analyzed for AS and DHA concentrations using a ThermoFinnigan TSQ mass spectrometer equipped with a CTC/PAL refrigerated auto-sampler. Drugs were chromatographically separated using an acetonitrile/ammonium acetate gradient through a Waters XTerra C_18_ column (2.1 × 50 mm, 3.5 μm particle size) and corresponding pre-column. Quantification was done using the most abundant daughter ions following positive electrospray ionization. Chromatograms were analyzed using Xcalibur mass spectrometry software. The responses-concentrations standard curves were fitted and weighted. Least square regression analysis was performed and the resulting parameters used to back calculate the concentration of the standard and calculate the concentration of the animal samples which are reported as ng/mL plasma.

### 3.6. Hematology and Recovery Studies

At 24 and 96 h after the final dose of Chinese and WRAIR intravenous AS, male and female animals were anesthetized by isoflurane inhalation anesthesia (Abbott Labs, North Chicago, IL, USA) delivered by a small-animal anesthesia system (Euthanex Corp, Palmer Park, PA, USA). Each animal was initially placed in a chamber primed with 5% isoflurane, and upon induction, maintained in a non-rebreathing mask at 2% isoflurane delivered with 0.5 mL O_2_ per minute. Animals were bled from the abdominal inferior vena cava after an abdominotomy. One mL of the blood sample was placed in K3 EDTA tubes (Beckton Dickinson, Franklin Lakes, NJ, USA) for CBC analysis using the ABX Pentra 60 hematology analyzer (ABX diagnostics, Irvine, CA, USA). Another 0.7 mL of blood was placed in serum separator tubes (Becton Dickinson, Franklin Lakes, NJ, USA) for biochemistry analysis using commercial assay kits on the Vitros 250 Chemistry analyzer (Johnson & Johnson, Rochester, NY, USA). Reticulocyte analysis was performed utilizing light microscopy. After sample collection, rats were euthanized by cervical dislocation.

### 3.7. Data Analysis

The concentration-time data for AS and DHA were fit to a non-compartment fitting procedure (WinNonlin 5.2, Pharsight Co. Mountain View, CA. USA). The area under the curve (AUC) was determined by the linear trapezoidal rule with extrapolation to infinity based on the concentration of the last time point divided by the terminal rate constant. The bioequivalence (BE) evaluation of the two formulations were analyzed by using BE software (WinNonlin 5.2, Pharsight Co. Mountain View, CA. USA) in rats for a NDA application. The WRAIR AS formulations tested were considered bioequivalent if the 90% confidence interval (CI) of the mean difference for each pharmacokinetic variable lies within 80% and 125% of the Chinese AS formulation.

## 4. Conclusions

The aims of this investigation were to assess the steady-state pharmacokinetic parameters of AS and its active metabolite, DHA, in 5% NaHCO_3_ (China) or 0.3 M PBS (WRAIR) formulations following single and multiple intravenous administrations, and to identify parameters that may affect their observed differences in bioequivalence in male rats. A series of blood samples were taken over a 72 h period by using an automated blood sampler, and drug concentrations were analyzed by LC-MS/MS with positive ion electrospray ionization using multiple reaction monitoring. The 90% CI of the difference between the two intravenous formulations was contained within 80–125% of the geometric mean of PK parameters after single and multiple doses for AS in all single and multiple administrations and for DHA in only multiple doses studies. The equivalent results exhibited that The bioequivalence standard was fulfilled in the two-period studies with the parent compound (AS) where the 90% CI of the differences in PK parameters for the two formulations fell within 82.3–117.7 for all parameters, and the bioequivalence standard was partially satisfied for DHA following single or multiple intravenous administrations in rats. The WRAIR AS formulation was considered to be bioequivalent to the Chinese formulation at steady-state according to the total drug exposure, in terms of both the parent drug and the active metabolite, extending over single and multiple administrations in rats treated in two-sequences and in two-period studies.

## Figures and Tables

**Table 1:** Pharmacokinetic parameters of artesunate (AS/NaHCO_3_, a Chinese formulation) and dihydroartemisinin (DHA), an active metabolite of AS, at dose of 20 mg/kg following single and multiple intravenous injection daily for three days in male rats in two-period (n = 6).

**PK Parameters**	**Period 1**		**Period 2**	

	**Parent Drug (AS)**	**Active metabolite (DHA)**	**Parent Drug (AS)**	**Active metabolite (DHA)**
Day 1				
C_max_ (ng/mL)	4300.4 ± 827.1	3361.4 ± 846.0	4139.4 ± 853.9	2689.1 ± 643.1
T_max_ (h)	0.083 ± 0.0	0.25 ± 0.0	0.083 ± 0.0	0.25 ± 0.0
AUC_0–3 h_	867.5 ± 136.7	1568.6 ± 346.6	932.6 ± 144.2	1223.4 ± 266.3
AUC_infininty_ (ng·h/mL)	871.5 ± 138.1	1569.5 ± 349.8	935.3 ± 143.9	1225.8 ± 265.7
t_1/2_ distribution (h)	0.06 ± 0.01	0.12 ± 0.02	0.08 ± 0.03	0.12 ± 0.02
t_1/2_ elimination (h)	0.40 ± 0.07	0.75 ± 0.10	0.43 ± 0.07	0.73 ± 0.17
Vss (liter/kg)	5.97 ± 1.27	-	5.70 ± 1.94	-
CL (mL/min/kg)	380.5 ± 54.2	-	359.8 ± 56.2	-
MRT (h)	0.26 ± 0.04	0.38 ± 0.13	0.26 ± 0.05	0.46 ± 0.14
Day 3				
C_max_ (ng/mL)	2464.8 ± 758.5	2779.4 ± 419.4	2542.6 ± 740.2	2452.1 ± 497.7
T_max_ (h)	0.083 ± 0.0	0.17 ± 0.09	0.083 ± 0.0	0.25 ± 0.0
AUC_0–3 h_	614.2 ± 191.8	990.8 ± 224.7	665.4 ± 236.3	905.2 ± 188.7
AUC_infininty_ (ng·h/mL)	616.9 ± 192.8	993.8 ± 224.8	667.2 ± 237.5	909.7 ± 187.7
t_1/2_ distribution (h)	0.10 ± 0.03	0.09 ± 0.01	0.12 ± 0.03	0.08 ± 0.01
t_1/2_ elimination (h)	0.41 ± 0.11	0.68 ± 0.15	0.39 ± 0.13	0.72 ± 0.12
Vss (liter)	8.67 ± 2.61	-	8.54 ± 4.27	-
CL (mL/min/kg)	570.2 ± 170.2	-	562.0 ± 240.4	-
MRT (h)	0.29 ± 0.10	0.35 ± 0.06	0.26 ± 0.09	0.38 ± 0.02
C_min_ (ng/mL)	4.35 ± 2.54	2.96 ± 2.31	3.35 ± 2.11	4.52 ± 2.25
C_av_ (ng·h/mL)	204.7 ± 63.9	330.2 ± 74.9	221.8 ± 78.8	301.8 ± 62.9
Degree of Fluctuation (%)	1216 ± 144	860.1 ± 121.1	1192 ± 219	844 ± 64
Total AUC (1–3D, ng·h/mL)	2206.5 ± 454.9	3825.7 ± 686.9	2383.9 ± 543.6	3206.1 ± 632.8
Ratio of AUC (D3/D1)	0.70 ± 0.15	0.65 ± 0.17	0.71 ± 0.17	0.75 ± 0.12
Ratio of C_max_ (D3/D1)	0.42 ± 0.08	0.87 ± 0.13	0.58 ± 0.29	1.03 ± 0.24
Ratio of DHA/AS (1–3D)		1.78 ± 0.43		1.46 ± 0.68

MRT = mean residence time, AS = artesunate, DHA = dihydroartemisinin, D = Day, C_av_ = Concentration Average = AUC_0–72 h/72_; DF = Degree of Fluctuation = 100% × (C_max_ − C_min_)/C_av_.

**Table 2:** Pharmacokinetic parameters of artesunate (AS/0.3M PBS Buffer, a WRAIR formulation) and dihydroartemisinin (DHA), an active metabolite of AS, at dose of 20 mg/kg following single and multiple intravenous injection daily for three days in male rats in two-period (n = 6).

**PK Parameters**	**Period 1**		**Period 2**	

	**Parent Drug (AS)**	**Active metabolite (DHA)**	**Parent Drug (AS)**	**Active metabolite (DHA)**
Day 1				
C_max_ (ng/mL)	4032.6 ± 1032.0	3153.8 ± 662.9	4209.2 ± 618.1	3521.7 ± 892.6
T_max_ (hr)	0.083 ± 0.0	0.25 ± 0.0	0.083 ± 0.0	0.25 ± 0.0
AUC_0–3 h_	879.4 ± 167.4	1459.7 ± 212.8	851.4 ± 115.9	1509.1 ± 293.0
AUC_infininty_ (ng·h/mL)	882.3 ± 167.2	1462.8 ± 211.1	854.5 ± 115.8	1514.6 ± 293.0
t_1/2_ distribution (h)	0.08 ± 0.02	0.12 ± 0.01	0.08 ± 0.01	0.14 ± 0.01
t_1/2_ elimination (h)	0.41 ± 0.03	0.98 ± 0.14	0.44 ± 0.06	0.91 ± 0.07
Vss (liter/kg)	6.89 ± 2.38	-	6.54 ± 1.15	-
CL (mL/min/kg)	377.6 ± 67.2	-	386.9 ± 52.1	-
MRT (h)	0.30 ± 0.06	0.39 ± 0.08	0.28 ± 0.04	0.40 ± 0.04
Day 3				
C_max_ (ng/mL)	2430.9 ± 713.5	2812.3 ± 529.4	2710.1 ± 759.7	3124.2 ± 612.4
T_max_ (hr)	0.083 ± 0.0	0.25 ± 0.0	0.083 ± 0.0	0.25 ± 0.0
AUC_0–3 h_	611.1 ± 162.1	1100.7 ± 177.6	669.7 ± 159.3	1041.7 ± 160.2
AUC_infininty_ (ng·h/mL)	613.2 ± 162.8	1110.9 ± 174.2	672.7 ± 160.3	1046.8 ± 160.0
t_1/2_ distribution (h)	0.10 ± 0.04	0.09 ± 0.01	0.14 ± 0.02	0.09 ± 0.14
t_1/2_ elimination (h)	0.47 ± 0.10	0.90 ± 0.12	0.54 ± 0.19	0.88 ± 0.14
Vss (liter)	9.10 ± 4.41	-	8.46 ± 3.13	-
CL (mL/min/kg)	558.7 ± 118.3	-	516.6 ± 161.9	-
MRT (h)	0.27 ± 0.09	0.34 ± 0.11	0.27 ± 0.06	0.39 ± 0.03
C_min_ (ng/mL)	3.56 ± 0.85	6.59 ± 3.06	3.87 ± 1.57	5.38 ± 3.77
C_av_ (ng·h/mL)	203.7 ± 54.0	367.4 ± 59.7	223.2 ± 53.1	347.1 ± 53.4
Degree of Fluctuation (%)	1194 ± 169	765 ± 97	1218 ± 172	900 ± 107
Total AUC (1-3D, ng·h/mL)	2244.7 ± 460.4	3869.2 ± 546.5	2286.3 ± 371.7	3832.7 ± 571.8
Ratio of AUC (D3/D1)	0.69 ± 0.12	0.76 ± 0.08	0.78 ± 0.14	0.71 ± 0.14
Ratio of C_max_ (D3/D1)	0.70 ± 0.17	1.09 ± 0.63	0.58 ± 0.16	0.95 ± 0.20
Ratio of DHA/AS (1–3D)		1.77 ± 0.41		1.70 ± 0.25

MRT = mean residence time, AS = artesunate, DHA = dihydroartemisinin, D = Day, C_av_ = Concentration Average = AUC_0–72 h/72_; DF = Degree of Fluctuation = 100% X (C_max_ − C_min_)/C_av_.

**Table 3:** The 90% confidence interval (CI) for the means of C_max D1_, C_max D3_, AUC_D1_, AUC_D3_, AUC_1-3D_, C_av_, and DF between 5% NaHCO_3_ solution (China) and 0.3 M PBS Buffer (WRAIR) formulations following single and multiple intravenous injection of artesunate (AS) at 20 mg/kg daily for three days in male rats. To establish bioequivalence (BE), the calculated CI should fall within a BE limit (90% CI) of 80–125% for the ratio of the product averages (in each group of formulation or period, n = 12).

**PK Parameters**	**Artesunate (AS, Parent drug) 90% CI for T/R**		**BE**	**Dihydroartemisinin (DHA, a metabolite of AS) 90% CI for T/R**		**BE**

	**Lower (%)**	**Upper (%)**		**Lower (%)**	**Upper (%)**	
Single Dose						
C_max D1_	90.21	109.79	Yes	75.17	124.78	No
AUC_D1_	88.72	111.28	Yes	82.04	117.96	Yes
Multiple Doses						
C_max D3_	83.68	116.32	Yes	81.94	118.06	Yes
AUC_D3_	82.30	117.69	Yes	71.32	128.67	No
AUC_D1–3_	87.65	112.35	Yes	80.11	120.89	Yes
C_av_	89.34	106.31	Yes	82.16	93.09	Yes
DF	98.46	100.87	Yes	99.09	106.55	Yes

BE = bioequivalence; T = Test article (WRAIR formulation); R = Reference article (China Formulation); C_av_ = Concentration Average = AUC_0–72 h/72_; DF = Degree of Fluctuation = 100% × (C_max_ − C_min_)/C_av_.;

* The data presented are calculated by WinNonlin 5.2 with BE program.

**Table 4:** Hematological results from male and female rats dosed with multiple injectable artesunate (AS) in Chinese and WRAIR formulations daily for 3 days at dosage of 20 mg/kg and sampled at day zero and day 3 after the final dose (n = 6).

**PK Parameters**	**Control s**	**Chinese AS IV Formulation**		**WRAIR AS IV Formulation**	

		**D1 after last dose**	**D4 after last dose**	**D1 after last dose**	**D4 after last dose**
Male rats					
RBC (× 10^6^/mm^3^)	6.6 ± 0.3	5.8 ± 0.1*	6.4 ± 0.5	6.1 ± 0.1	6.2 ± 0.7
WBC (× 10^3^/mm^3^)	11.8 ± 2.8	13.2 ± 3.1	11.4 ± 3.0	12.6 ± 3.4	11.9 ± 2.4
HCT (%)	39.3 ± 1.1	35.5 ± 2.7	36.7 ± 4.1	37.1 ± 3.8	37.2 ± 4.2
Hb (g/dL)	13.4 ± 0.4	12.3 ± 0.6*	13.8 ± 1.1	11.9 ± 0.7*	13.6 ± 0.8
Retic (% of RBC)	13.3 ± 4.7	4.0 ± 2.6**	16.2 ± 4.3	3.8 ± 2.3*	14.4 ± 3.7
Female rats					
RBC (× 10^6^/mm^3^)	6.2 ± 1.1	5.8 ± 0.4	5.9 ± 0.6	6.1 ± 0.5	6.4 ± 0.8
WBC (× 10^3^/mm^3^)	8.7 ± 0.8	11.9 ± 0.6*	10.8 ± 0.4*	13.2 ± 0.9*	10.1 ± 0.9
HCT (%)	34.9 ± 5.3	33.1 ± 1.2	33.7 ± 1.5	34.2 ± 1.6	33.8 ± 2.3
Hb (g/dl)	12.8 ± 2.2	12.4 ± 0.6	12.7 ± 0.8	11.8 ± 0.5	13.1 ± 0.9
Retic (% of RBC)	8.3 ± 4.5	0.5 ± 0.3**	9.2 ± 2.2	1.3 ± 0.7 *	10.3 ± 3.6

a Values are means, S.D. in parentheses;

* significantly different to control animals, *P* < 0.05;

** *P* < 0.01;

b Abbreviations units: RBC = red blood cells, × 10^6^/mm^3^; HCT = hematocrit, %; Hb = Hemoglobin, g/dL; Retic = absolute reticulocyte count, % of RBC; WBC = white blood cells, × 10^3^/mm^3^; D1 = Day 1; D4 = Day 4.
